# Routine radiographs at time of pin removal after closed reduction and percutaneous pinning for type 2 supracondylar humerus fractures do not change management: a retrospective cohort study

**DOI:** 10.1007/s11832-016-0744-6

**Published:** 2016-06-06

**Authors:** Sumeet Garg, Nikki Bloch, Micaela Cyr, Patrick Carry

**Affiliations:** Pediatric Orthopaedics and Spine Surgery, Orthopaedics Institute, Children’s Hospital Colorado, 13123 East 16th Avenue, Box 060, Aurora, CO 80045 USA; Musculoskeletal Research Center, Orthopaedics Institute, Children’s Hospital Colorado, Aurora, Colorado USA

**Keywords:** Supracondylar humerus fracture, Radiographs, Pin removal, Type 2, Pediatric

## Abstract

**Purpose:**

Radiographs are usually taken on day of pin removal for children treated with closed reduction and percutaneous pinning (CRPP) of type 2 supracondylar humerus fractures. The purpose of this study was to determine whether radiographs taken at time of pin removal for patients recovering uneventfully alter management.

**Methods:**

After IRB approval, billing records identified 1213 patients aged 1–10 years who underwent elbow surgery between 2007 and 2013 at our institution for a supracondylar humerus fracture. Of these patients, 389 met inclusion criteria. Clinical charts were reviewed for demographics, operative details, and clinical follow-up, focusing on clinical symptoms present at pin removal. Radiographs taken at time of pin removal and subsequent visits were assessed for healing and fracture alignment.

**Results:**

In *no* case was pin removal delayed based on radiographs. One hundred and nineteen (31 %) patients had radiographs taken following pin removal; in no case was loss of reduction found among these patients. No cases of neurologic or vascular injury, re-fracture, or loss of reduction occurred. Infection occurred in 12 patients (3 %). Pins were kept in place for 23.8 ± 4.4 days. Eighty-six patients (22 %) had additional intervention after pin removal (cast application in all cases). Of 389 patients, 75 (19 %) had no documented reason for extended casting, four (1 %) were extended based on physician evaluation of radiographs, and seven (2 %) were extended for other reasons.

**Conclusions:**

Elimination of radiographs at time of pin removal should be considered. If continuing to obtain radiographs at pin removal, we recommend removing pins before taking radiographs to reduce patient fear and anxiety from visualizing percutaneous pins.

## Introduction

At our institution, type 2 supracondylar humerus fractures are almost exclusively treated operatively with closed reduction and percutaneous pinning (CRPP). This management strategy has low rates of malunion, nerve injury, vascular injury, or compartment syndrome in patients and avoids the high rate of loss of reduction observed with non-operative care [1–4]. Intra-operative fluoroscopy is routinely used to assess fracture reduction and verify appropriate divergent placement of percutaneous pins. Prior studies have demonstrated extremely low rates of loss of reduction or implant failure in patients with supracondylar humerus fractures treated by CRPP [5–8].

Recent literature has demonstrated an interest in reducing the instances of unnecessary radiographs. Two studies have suggested that radiographs after surgical treatment but prior to planned pin removal do not alter management [6, 9]. The purpose of this study was to evaluate the utility of post-operative radiographs taken at time of pin removal for patients healing uneventfully after CRPP of a type 2 supracondylar humerus fracture. We hypothesized that the interventions at time of pin removal based on radiographic findings would be rare.

## Materials and methods

**Fig. 1:**
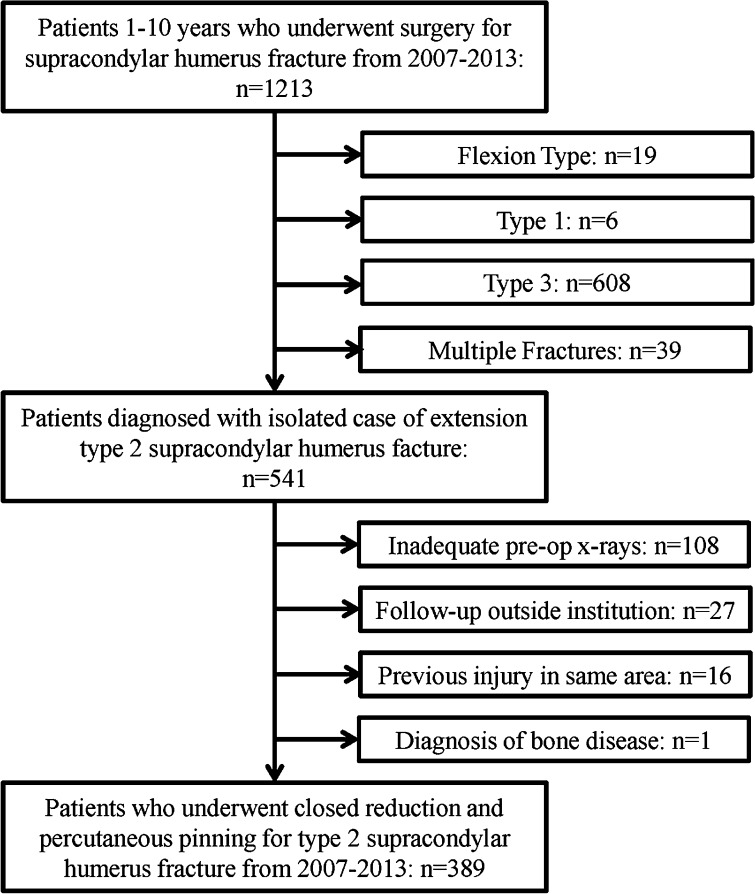
Description of the study cohort

Following institutional review board approval, our institutional billing database was queried for children aged 1–10 years who had surgery for supracondylar humerus fractures between 2007 and 2013. Inclusion criteria for the cohort were patients who had CRPP for an extension type 2 supracondylar humerus fracture. Exclusion criteria were children with previous surgery or fracture at the distal humerus, children following up outside our institution for pin removal, children with metabolic bone diseases, and/or children with inadequate pre-operative imaging. Pre-operative radiographs were reviewed to establish diagnosis of extension type 2 fractures based on the Gartland classification [10]. Development of the study cohort is shown in Fig. 1.

Study data were retrospectively collected including demographics (age and gender), surgical characteristics (attending surgeon, number of pins, pin configuration, and pin size), and post-operative clinical characteristics (days to pin removal, if pin removal was early or delayed, neurologic complication, vascular complication, surgical site infection, re-fracture after pin removal, loss of reduction on radiographs at pin removal and at subsequent visits, total number of radiographs taken post-operatively). Early pin removal is defined as pins removed earlier than planned at time of surgery. Eighteen surgeons were included in the study cohort. They had varied regimens in terms of need for casting after pin removal and number of visits scheduled after pin removal. In general, first post-operative follow-up occurs 2–3 weeks after surgery for pin removal. Follow-up after pin removal is on an as needed basis. Additional intervention after pin removal due to radiographic findings was the primary outcome variable of interest.

Radiographs at day of pin removal and radiographs taken at any subsequent visits were compared to pre-operative and intra-operative imaging to discern loss of fracture reduction and the presence of fracture healing. Visible fracture callus adjacent to the cortices on both frontal and lateral views was considered appropriate fracture healing. The lead author reviewed all images and was not involved in patient care for this population. The senior author was a surgeon for patients in the cohort and assisted with radiographic review in selected cases. All were de-identified prior to review by the senior author.

Descriptive statistics were used to summarize the proportion of subjects that underwent additional interventions after pin removal and the reason(s) for the interventions. A two-sided binomial test was used to test the null hypothesis that the proportion of subjects that underwent intervention secondary to radiographic findings was equal to 5 %.

## Results

The median number of total post-operative radiographs taken was four (range 2–14) while the median number of radiographs taken on day of pin removal was two (range 2–4). Of 389 patients, 119 (31 %) had radiographs taken after day of pin removal during follow-up visits. The median of number of radiographs following pin removal for these 119 patients was two (range 2–12). The only intervention encountered after pin removal was extending immobilization in cast. Following pin removal, 86/389 (22 %) patients were immobilized for additional time (1–4 weeks). Specific reasons for extended immobilization included the following: unknown reason 75/389 (19.28 %), early pin removal 1/389 (0.26 %), pain/tenderness 3/389 (0.77 %), family or physician concern due to an active child 3/389 (0.77 %), and surgeon interpretation of delayed healing from radiographs 4/389 (1.03 %). Overall, the proportion of subjects that underwent a change in post-operative care based on radiographic findings (surgeon interpretation of delayed healing) was significantly less than 5 % [*p* = 0.0003]. Retrospective review of radiographs at time of pin removal by the current research team found no cases where there was loss of reduction compared to intra-operative fluoroscopy images. Healing facture callus was found on all radiographs at time of pin removal. Additionally, for the 119 patients who had radiographs taken after pin removal, no loss of reduction was identified after pin removal.

Complications included infection in 12 (3 %) patients and referral to physical or occupational therapy in 10 (3 %) patients. Six patients required return to the operating room for debridement of infection, and six patients had infection treated with antibiotics only. These cases all showed appropriate alignment of fracture and callous formation on review of the postoperative radiographs. Two patients returned to the operating room for pin removal. Seven (2 %) patients complained of pain in the elbow at time of pin removal; the remainder were not symptomatic at time of pin removal. No neurologic injury, vascular injury, or loss of reduction occurred in the cohort.

## Discussion

Radiographs on day of planned pin removal after CRPP for type 2 supracondylar humerus fractures consistently showed stable fracture alignment and visible fracture callus in this cohort. Complication rates reported after CRPP in this cohort are consistent with previously published studies [1, 4]. Despite 22 % of children having casts applied after pin removal, there was no instance where pin removal was delayed at the 3–4 week post-operative visit because of radiographic findings. The vast majority of children having extended immobilization after pin removal appeared to be individual surgeon preference.

**Fig. 2:**
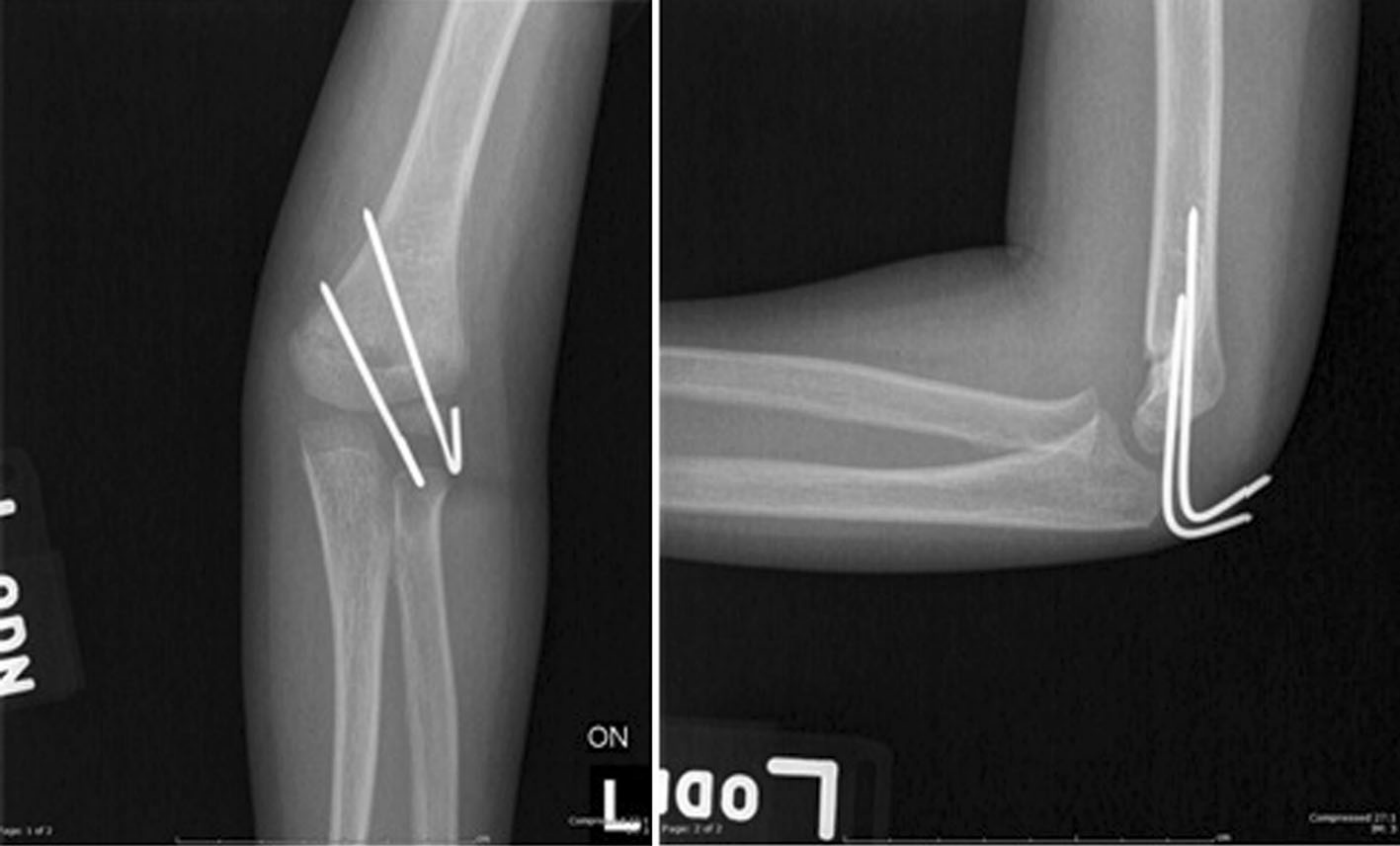
Example of radiograph at time of pin removal for a patient whose immobilization was extended based on surgeon interpretation of radiographic findings

We identified only four (1 %) subjects where a surgeon documented their assessment of poor fracture healing on radiographs; in no other instance was the radiographic appearance of the humerus at time of pin removal felt to alter management. In these cases, the radiographs at pin removal do not show any obvious evidence of delayed healing or fracture malalignment based on review of de-identified images by the senior author. An example of one of these cases is shown in Fig. 2. Notably, pin removal was not delayed in these cases, rather immobilization was extended. Consistent with our hypothesis, the proportion of subjects that required post-operative care modifications on the basis of radiographic findings was significantly less than 5 %.

Limitations to this study include variability in the post-operative management of patients following CRPP among the 18 treating surgeons, especially in regards to extended immobilization. Reviewing charts retrospectively, it was not possible to confirm that decisions regarding the need for extended immobilization were primarily based on surgeon preference. However, in speaking to the two surgeons who immobilized the majority of their patients after pin removal, it was clear that they generally prefer to place children in a rigid cast following pin removal [11]. Although there was no documented case of re-fracture in this cohort, most children did not follow-up after pin removal, as is standard clinical practice at our institution, and potentially may have sought treatment at another institution if they had a repeat fracture.

Given the limitations of this study and high number of patients receiving additional intervention after pin removal, we cannot definitively support elimination of radiographs at time of planned pin removal. Nevertheless, it was very clear that the findings on radiographs did not alter the plan for pin removal at the 3–4 week time point after surgery. A study by Schlecter and Dempewolf on supracondylar humerus fractures revealed similar results. Pin removal occurred, on average, 27 days after surgery, similar to the average of 23 days exhibited in this study. In no case was pin removal delayed, and no complications were noted after pin removal. Patients in their study cohort, however, did not have cast immobilization extended after pin removal. While Schlecter and Dempewolf’s cohort included type 2, type 3, and flexion type supracondylar humerus fractures, the results of their study similarly indicate that radiographic findings do not alter the plan for pin removal [11].

Pin removal, though a relatively painless procedure, does induce significant fear and anxiety for the patient [12, 13]. Limiting the amount of time a patient has to visualize the pins may reduce this fear and anxiety and, by extension, reduce the perceived pain of the procedure [14]. The patient experience at day of planned pin removal may be enhanced if the pins were rapidly removed after the post-operative immobilization (cast or splint) is removed in the office *prior* to proceeding for radiographic evaluation [11].

Radiographic analysis did not identify any impaired fracture healing or delay in planned pin removal in this cohort of healthy children with type 2 supracondylar humerus fractures at 3–4 weeks post-surgery. For patients who had radiographs taken after pin removal, these images showed continued stable alignment and no evidence of loss of reduction. Elimination of radiographs at time of planned pin removal would reduce cost and radiation exposure to patients while also improving clinic efficiency. Consideration should be made to eliminate completely radiographs on day of planned pin removal for children who are asymptomatic. Based on our results, more research is necessary to definitively support elimination of these radiographs; however, to minimize patient and family fear and anxiety pin removal should occur immediately after removing post-operative immobilization if continuing to order radiographs at day of planned pin removal. Current practice by the senior author is to remove pins at approximately 3 weeks post-operation and obtain a frontal and lateral view of the injured elbow after removal of pins.
